# A gene based combination test using GWAS summary data

**DOI:** 10.1186/s12859-022-05114-x

**Published:** 2023-01-03

**Authors:** Jianjun Zhang, Xiaoyu Liang, Samantha Gonzales, Jianguo Liu, Xiaoyi Raymond Gao, Xuexia Wang

**Affiliations:** 1 Department of Mathematics, University of North Texas, 225 Avenue E, Denton, TX 76201 USA; 2 Department of Epidemiology and Biostatistics, Michigan State University, 909 Wilson Rd Room B601, East Lansing, MI 48824 USA; 3 Department of Ophthalmology and Visual Science, Department of Biomedical informatics, Division of Human Genetics, Ohio State University, 915 Olentangy River Road, Columbus, OH 43212 USA; 4 Department of Biostatistics, Robert Stempel College of Public Health and Social Work, Florida International University, 11200 SW 8th street, Miami, FL 33174 USA

**Keywords:** Combination test, Score test, Burden test, Weighted sum of squared score test, Weighted sum statistic

## Abstract

**Background:**

Gene-based association tests provide a useful alternative and complement to the usual single marker association tests, especially in genome-wide association studies (GWAS). The way of weighting for variants in a gene plays an important role in boosting the power of a gene-based association test. Appropriate weights can boost statistical power, especially when detecting genetic variants with weak effects on a trait. One major limitation of existing gene-based association tests lies in using weights that are predetermined biologically or empirically. This limitation often attenuates the power of a test. On another hand, effect sizes or directions of causal genetic variants in real data are usually unknown, driving a need for a flexible yet robust methodology of gene based association tests. Furthermore, access to individual-level data is often limited, while thousands of GWAS summary data are publicly and freely available.

**Results:**

To resolve these limitations, we propose a combination test named as OWC which is based on summary statistics from GWAS data. Several traditional methods including burden test, weighted sum of squared score test [SSU], weighted sum statistic [WSS], SNP-set Kernel Association Test [SKAT], and the score test are special cases of OWC. To evaluate the performance of OWC, we perform extensive simulation studies. Results of simulation studies demonstrate that OWC outperforms several existing popular methods. We further show that OWC outperforms comparison methods in real-world data analyses using schizophrenia GWAS summary data and a fasting glucose GWAS meta-analysis data. The proposed method is implemented in an R package available at https://github.com/Xuexia-Wang/OWC-R-package

**Conclusions:**

We propose a novel gene-based association test that incorporates four different weighting schemes (two constant weights and two weights proportional to normal statistic ***Z***) and includes several popular methods as its special cases. Results of the simulation studies and real data analyses illustrate that the proposed test, OWC, outperforms comparable methods in most scenarios. These results demonstrate that OWC is a useful tool that adapts to the underlying biological model for a disease by weighting appropriately genetic variants and combination of well-known gene-based tests.

**Supplementary Information:**

The online version contains supplementary material available at 10.1186/s12859-022-05114-x.

## Background

To date, genome-wide association studies (GWAS) have identified more than thousands of genetic variants associated with complex traits or diseases. However, these identified genetic variants only can explain a small to modest fraction of heritability [1]. To identify genetic variants which can explain the missing heritability, people need to use data with larger sample size and/or more powerful statistical tests, especially when causal genetic variants have weak effects on complex traits. In reality, it is often difficult to access patients data directly, and thus difficult to obtain data with sufficiently large sample size. On the other hand, thousands of GWAS summary data are publicly and freely available. These GWAS data including *p*-values, effect sizes, directions of effects, or estimated statistics for single nucleotide polymorphisms (SNPs) motivate us to develop novel powerful methods for further analysis of GWAS summary data. The gene-based association test using GWAS summary statistics can be viewed as a complementary approach to the traditional single marker association test in GWAS.

When testing for genetic associations with a gene-based test, proper weights can boost power substantially. However, one major limitation of existing gene-based association tests lies in using weights predetermined biologically or empirically. This limitation often attenuates the power of a test. For example, both the burden test [2, 3] and the weighted sum of squared score (SSU) test [4] are typical combination methods. The burden test sets the same weight for each genetic variant, while the SSU test uses the Z-score as a weight for each genetic variant. The presence of non-associated SNPs in a gene can diminish the power of a test dramatically if an effective SNP selection method or weighting method is not adopted [5]. The SSU method is robust and powerful when there are protective, risk, and null variants in a considered region, but it is less powerful than the burden test when a large number of genetic variants in the considered region are causal and the direction of effects are the same. A statistical challenge is that the true association patterns are usually unknown. A test may perform well for one real dataset, but it may be less powerful for another dataset. There is no uniformly most powerful test which is powerful in every situation [6]. In this study, we intend to develop a test which is more powerful than well-known existing methods in most situations.

The power of a gene-based test depends on the underlying genetic architecture of a complex trait. For different traits, the genetic architecture can differ in number, location, effect size, and direction of effect for causal genetic variants in different genes. To circumvent the difficulties in gene-based association test, we propose the combination method, which is a general, flexible, and powerful method. When testing for weak associations caused by small effect sizes or low frequency common genetic variants, the proposed method performs significantly better than several popular gene based tests such as sum test (ST) [7], squared sum test (S2T) [7], adaptive test (AT) [7], adaptive sum of powered score tests (aSPU) [6], Gene-based Association Test using extended Simes procedure (GATES) [8] and sumSTAAR method which provides a framework for combining a wide range of gene-based association tests using summary statistics [9].

Testing association between a phenotype and a gene based on individual level data (i.e. genotypes) of genetic variants in the gene is the same as testing association between the phenotype and the gene based on summary statistics (i.e. Z-scores) in that gene [10, 11]. In the Methods section, we illustrated this conclusion with a score test framework. Furthermore, we proposed a new score test $S_s$ which can reach its maximum when weights of genetic variants is $Z^{'} R^{-1}$ where *Z* is the Z score summary statistics and R is the correlation matrix of genetic variants. Six existing methods can be considered as its special cases and are summarized in Table 1. As indicated in Table 1, six gene-based association tests based on individual level data can be easily modified to gene-based association tests based on GWAS summary data [11]. Based on the score test and other three typical methods, we propose a novel and powerful gene-based association test using GWAS summary data, named as OWC, which can reaching its maximum through finding the appropriate weights for the combination of the four tests. The burden test, SSU, weighted sum statistic (WSS) [13], and score test are special cases of the proposed OWC method. Furthermore, we show that OWC is more powerful than other comparison methods in most simulation studies and identifies more trait associated genes in three real datasets.

**Table 1:** Summary of the proposed score test $S_s$ and its special cases

	Method	Description	Weight	Test Statistic
General method	Score Test $S_s$	Maximum of the weighted sum of Z scores	$\varvec{W }=\varvec{ Z}^{\varvec{'}} \varvec{R}^{\varvec{-1}}$	$S_{s} = \varvec{Z}^{\varvec{'}}\varvec{R}^{\varvec{-1}}\varvec{Z}$
Special cases of the score test $S_s$	Special case 1: Sum of Squared Score Statistic (SSU)	Weighted sum of Z scores, weights are Z scores	$\varvec{W =R^{-1}Z, R=I}$	$S_{Q} = \varvec{Z^{'}Z}$
	Special case 2: SNP-set (Sequence) Kernel Association Test (SKAT)	Weighted sum of Z scores, weights are weighted Z scores	$\varvec{W =R^{-1}Z}, \varvec{R}=diag(a_{1}, \ldots , a_{m}), a_{m} \sim beta(1,25)$	$S_{SKAT} = \varvec{Z^{'}R^{-1}Z}$
	Special case 3: PathSPU(2)	Weighted sum of Z scores, weights are eQTL weighted Z scores	$\varvec{W =R^{-1}Z}, \varvec{R}=diag(a_{1}, \ldots , a_{m}), a_{m}$ are gene derived weights	$S_{pathSPU(2)} = \varvec{Z^{'}R^{-1}Z}$
	Special case 4: Sum of Powered Score (SPU): Data-adaptive weighted combination test.	Weighted sum of Z scores, weights are function of Z scores	$\varvec{W} = \varvec{Z}^{\gamma - 1}$	$SPU(\gamma ) = \sum _{m=1}^{M}\varvec{Z}_m^{\gamma }, \gamma = 1,2,\ldots ,8,\infty$
	Special case 5: Burden test	Weighted sum of Z scores, weights are all 1s	$\varvec{W} = (1, \ldots , 1)^{'}$	$L_B = L(1, \ldots , 1) = \sum _{m=1}^{M}{\textbf{Z}}_{\textbf{m}}$
	Special case 6: Weighted Sum Statistic	Weighted sum of Z scores, weights are related to MAFs	$\varvec{W} = (\frac{1}{\sqrt{p_1(1-p_1)}}, \ldots , \frac{1}{\sqrt{p_m(1-p_m)}})^{'}$, where $p_m$ is the MAF	$L_W = L(\frac{1}{\sqrt{p_1(1-p_1)}}, \ldots , \frac{1}{\sqrt{p_m(1-p_m)}}) = \sum _{m=1}^{M}\frac{1}{\sqrt{p_m(1-p_m)}}\cdot {\textbf{Z}}_{\textbf{m}}$

To evaluate the performance of the proposed method, we have conducted extensive simulation studies and real data analyses. We compared our method, OWC, with six existing comparable methods: (1) sum test (ST) [7]; (2) squared sum test (S2T) [7]; (3) adaptive test (AT) [7]; (4) adaptive sum of powered score tests (aSPU) [6]; (5) Gene-based Association Test using extended Simes procedure (GATES) [8]; and (6) sumSTAAR [9]. All of the comparison methods are designed for testing associated genes for a single trait. ST can be considered as a burden test statistic [12], S2T can be considered as a quadratic test similar as the SNP-set kernel association test (SKAT) [13], and AT is a combination of burden and quadratic tests, which is equivalent to the SKAT-O test [14]. The aSPU method chooses the most powerful test from a group of tests. GATES adopts an extended Simes procedure and uses GWAS summary statistics to correct for multiple testing issues and estimate the *p*-value promptly. sumSTAAR creates a frameworks to combine multiple gene based tests with ACAT method [15].

Our proposed method OWC is more powerful than the six comparable tests in most of the simulation scenarios. We further applied OWC and the other six tests to real datasets: (1) the GWAS summary data of schizophrenia (SCZ), which was obtained from the Psychiatric Genomics Consortium (PGC); (2) the GWAS meta-analysis summary data for fasting glucose, obtained from the European DIAMANTE study (a component of the UK Biobank). The results of the real data analyses demonstrate that OWC is the most effective test as it identified more trait-associated genes than other methods.

## Results

### Comparison of methods

The performance of the proposed method OWC are compared with six existing gene-based association tests: the sum test (ST), the squared sum test (S2T), adaptive test (AT) proposed by Guo and Wu [7], the adaptive sum of powered score tests (aSPU) method proposed by Kwak and Pan [6], the Gene-based Association Test that uses Extended Simes procedure (GATES) proposed by Li et al. [8], and the sumSTAAR [9].

Consider a gene with M genetic variants. Assume GWAS summary statistics such as Z scores are available for all the genetic variants in the gene. Denote $Z_m, m=1,2, \ldots ,M$ as the Z score of the *m*th variant. The six methods for testing genetic association are described briefly as follows:

1. Sum test (ST), $B=\sum _{m=1}^MZ_m$, which is similar as the burden test [12].
2. Squared sum test (S2T), $Q=\sum _{m=1}^MZ_m^2$, which is a special case of the SKAT method [13]. The squared sum test (S2T) is equivalent to the weighted sum of squared score (SSU) statistic [4].
3. Adaptive test (AT), $T=\min _{\rho \in [0,1]}P(Q_{\rho })$, where $Q_{\rho }=(1-\rho )Q+\rho B^2$, $P(Q_{\rho })$ denotes the corresponding *p*-value.
4. Adaptive sum of powered score tests (aSPU), aSPUs= $\min _{\gamma \in \Gamma }P_{SPUs(\gamma )}$, where $SPUs(\gamma )=\sum _{m=1}^MZ_m^{\gamma }$,where ${\gamma }$ is an integer.
5. Gene-based association test that uses extended Simes procedure (GATES), $p_{GATES}=\min \big (\frac{m_ep_{(j)}}{m_{e(j)}}\big )$, where $p_{(j)}$ is the $j^{th}$ smallest *p*-value, $m_{e(j)}$ is the effective number of independent *p*-values among the top *j* SNPs, $m_e$ is the effective number of independent *p*-values among the total M SNPs.
6. sumSTAAR combines *p *values of burden test, SKAT, SKAT-O [14], aggregated Cauchy association test (ACAT-V) [15], the tests using functional linear regression model (FLM) and principal component analysis (PCA) with ACAT method [15].

Denote ${\varvec{Z}}\thicksim \textrm{MVN}({\varvec{0}}, \varvec{R})$ where *R* is the linkage disequilibrium (LD) matrix of the gene, $B=1_M^{'}{\varvec{Z}}\thicksim N(0,1_M^{'}\varvec{R}1_M)$, where $1_M$ denotes a column vector of length *M* with elements 1s. $\frac{B^2}{1_M^{'}\varvec{R}1_M}$ follows $\chi _1$ distribution. The squared sum test Q=${\varvec{Z}}^{'}{\varvec{Z}}$ asymptotically follows $\chi ^2$ distribution which is equivalent to the weighted sum of independent $\chi _1$ distributed random variables where the weights are the eigenvalues of $\varvec{R}$. The *p*-value of the adaptive test *T* can be efficiently and simply computed by employing a one-dimensional numerically search over $\rho \in (0,0.01,0.04,0.09,0.16,0.25,0.5,1)$ following Wu et al. [16]. The three test ST, S2T, and AT can be obtained using the “sats” function in the “mkatr” package in R. Monte Carlo simulations are used to obtain the *p*-value of aSPU which can be obtained using the “aSPUs” function in the “aSPU” R package. The GATES method can be obtained from “gates” function in the “COMBAT” R package. sumSTAAR can be obtained from the sumFREGAT package (function sumSTAAR() in sumFREGAT v.1.2.3). When using the sumSTAAR method, we set the tests argument as the default tests - burden test, SKAT, and ACAT.

### Simulation studies

We conducted extensive simulation studies to evaluate the performance of the proposed method OWC. Following the simulation settings in Guo and Wu [17], we performed the type I error and power comparisons between OWC and the six comparable methods. Estimating LD among genetic variants using any reference data from the same ancestry is mostly accurate with an estimated inflation factor close to 1 [6]. Because of this, we estimated the LD between genetic variants in a gene using the haplotypes with ancestry from northern and western Europe (CEU) obtained from the 1000 Genomes project [18].

#### Type I error

To evaluate the type I error, we obtain similar ${\varvec{Z}}$ scores as in GWAS summary data from a multivariate normal distribution $\textrm{MVN}({\varvec{0}}, \varvec{R})$, where $\varvec{R}$ denotes the corresponding LD matrix of gene *EPB*41. Gene *EPB*41 colocalizes with *AMPA* receptors which is thought to interact with the cytoskeleton [19]. Abnormalities of brain-region in the expression of subunits of the *AMPA* subtype of glutamate receptors in Schizophrenia patients have been identified [20]. As in real data analysis, we first remove rare variants on gene *EPB*41 from our analysis and keep 11 SNPs with minor allele frequency (MAF) in the range from 0.067 to 0.453 in the simulation studies. The LD matrix $\varvec{R}$ of gene *EPB*41 is estimated by using the 1000 Genomes Project reference panel [18]. Additional file 1: Linkage disequilibrium matrix of gene *EPB41*. Fig. S1 shows the LD matrix with pairwise correlations for the 11 SNPs in *EPB*41. Coefficients of five pairwise LD ($r^2$) are greater than 0.5, and the others’ are less than 0.5. We use [21] to simulate the effect size beta of a causal genetic variant and its standard error for the sumSTAAR method. To mimic the real schizophrenia data used in Real Data Analysis section, we use the numbers of cases as 13,833 and the number of controls as 18,310 as inputs to the $simulated\_vbeta$ function and adjust “gamma.W” based on various simulation scenarios. We evaluated the proposed method by using five different significance levels: $\alpha =10^{-3},10^{-4},10^{-5}$, $2.5\times 10^{-6}$ and $2.80\times 10^{-6}$. In the simulations, *p*-values of the proposed method and aSPU are estimated with 10^7^ times replications. The type I error rates are estimated based on 10^7^ replications. Table 2 shows that the type I error rates of all of the methods are well controlled except that there is slight type I inflation of the sumSTAAR method.

**Table 2:** Ratio of estimated type I error rates by the significance level for different test methods

*α*-level	ST	S2T	AT	GATES	aSPU	sumSTAAR	OWC
1 × 10^−3^	1.02	1.02	1.02	1.04	1.03	0.86	1.01
1 × 10^−4^	1.00	1.03	1.00	1.03	1.02	1.10	1.04
1 × 10^−5^	1.00	1.04	0.99	1.05	1.08	2.50	1.00
2.5 × 10^−6^	1.04	1.14	1.00	0.92	1.04	3.00	1.08
2.8 × 10^−6^	1.10	1.07	0.97	1.00	1.01	3.57	1.02

Notes: The comparison methods sum test(ST), squared sum test (S2T) and adaptive test (AT) mentioned in the paper are equivalent to the methods burden test ($L_B$), the sum of squared score test ($S_Q$) and the combination of $L_B$ and $S_Q$, respectively. Let $S_S$ denote the score test and $L_W$ denote the weighted sum statistic. The proposed combination method OWC is a combination of $L_B$, $L_W$, $S_S$, and $S_Q$. sumSTAAR is a fexible framework for gene-based association studies using GWAS summary statistics

#### Power analysis

We further conduct extensive simulations to evaluate the power of the proposed method. We consider different scenarios in terms of different number of causal genetic variants, effect sizes and directions of causal variants, different number of SNPs, LD structure, and allele frequency spectrum of the considered region. Gene *EPB*41 contains 11 common SNPs. The range of the minor allele frequencies is (0.067, 0.453). The coefficients of five pairwise LD ($r^2$) are greater than 0.5 in *EPB*41 (Additional file 1: Linkage disequilibrium matrix of gene *EPB41.* Fig. S1). We simulate 10^4^ summary statistics from $\textrm{MVN}(\varvec{A}\times \triangle , \varvec{R})$ where $\varvec{A}$ denotes the directions of effects of causal variants (i.e. risk or protective effect), $\triangle$ denotes different settings of the effect sizes of causal variants. $\varvec{R}$ denotes the corresponding LD matrix of *EPB*41. We randomly select a number of SNPs (e.g. 2, 3, 4, or 5) as causal variants from *EPB*41. For a given gene, we randomly set the effects of the causal variants by drawing the corresponding number of elements of $\varvec{A}$ equal to 1 or − 1, and set the effects of other variants as 0. Table 3 shows the estimated power under three combinations of $\varvec{A}$ for different settings of $\triangle$: a set of fixed values of $\triangle$, two randomly simulated $\triangle$ where one is from uniform distribution, and the other from normal distribution. We use $2.50\times 10^{-6}$ as the significance level to claim a significant finding.

Table 3 shows that the proposed method OWC performs robustly well across all scenarios. It has the highest power in almost all of the scenarios when compared to the six other tests demonstrated in Table 3. The advantage of OWC may be attributed to the fact that it is an ultimately derived test after incorporating two kinds of burden tests and two kinds of quadratic tests. Among the four gene-level test statistics in OWC, the score test ($S_s$) that we proposed is to find the appropriate weights for genetic variants which allows the score statistic reaches its maximum. The power gained of OWC may be from two types of maximization in our proposed method: 1) to find the appropriate weights for the four gene-level tests in the combination to let the combination to reach its maximum; 2) to find the appropriate weights for genetic variants in the considered gene to let $S_s$ to reach its maximum. Therefore, the OWC test can reach the largest power. When $\triangle$ uses the settings of the fixed values, the power of the S2T and GATES methods increases as the effect size increases ($\triangle$ = (4,2,1) vs.(8,4,2) when *A*=(1,1,1), (1,1,− 1), or (1,− 1,− 1)). When $\triangle$ = (4,2,1), the powers of S2T and GATES are extremely low no matter *A* = (1,1,1), (1,1,− 1), or (1,− 1,− 1). This implies that their powers may suffer significant losses compared to the other methods when there are weak genetic effects. S2T and GATES are all robust to the direction of effects among causal SNPs since S2T is a quadratic method and GATES is a *p*-value combination method. When $\triangle$ = (8,4,2), the powers of S2T and GATES are high no matter *A* = (1,1,1), (1,1,− 1), or (1,− 1,− 1). When one or two causal variants have weak protective effects and the other causal variant has medium risk effect, all of the methods are significantly less powerful except for OWC ($\triangle$ = (4,2,1) when *A* = (1,1,− 1), or (1,− 1,− 1)). This conclusion is verified by the results from the normal distribution settings of $\triangle$. The results of a uniform distribution settings of $\triangle$ confirm that the power of the burden test ST is attenuated when there are different directions of effects of causal variants. Both AT and aSPU are adaptive methods by combining the burden test and quadratic test methods together, suffering a relatively small power loss when there are weak and different directions of effects. The power of a method increases as the number of risk causal variants increases. For example, when we keep two protective causal variants and increase the number of risk causal variants from 1, to 2, and then 3 ($\triangle$ = (4,2,1) and *A* = (1,− 1,− 1), $\triangle$ = (4,4,2,1) and *A* = (1,1,− 1,− 1), $\triangle$ = (4,4,4,2,1) and *A* = (1,1,1,− 1,− 1)), the power of all of the methods increases. sumSTAAR is less powerful than the GATES method when $\triangle$ uses the settings of the uniform and normal distributions but sumSTAAR is more powerful than GATES in some of situations when $\triangle$ uses the settings of the fixed values. In summary, our proposed test OWC is robust and powerful regardless of whether the causal genetic variants in a gene have the same or different directions of effects, especially when weak effect sizes exist.

**Table 3:** Power comparison between OWC and the other six tests. Data are simulated from $N(\varvec{A}\times \triangle , \varvec{R})$. $\varvec{A}$ has three or four nonzero elements with different signs which represent whether the causal variants are risk or protective. $\triangle$ denotes the different settings of effect sizes. R is the corresponding LD matrix of gene *EPB*41. Power (%) is estimated under $2.5\times 10^{-6}$ significance level

No. causal variants	nonzero $\triangle$	nonzero $\varvec{A}$	AT	S2T	ST	GATES	aSPU	sumSTAAR	OWC
3	U(1,5)	(1,1,1)	85.0	35.0	86.0	55.2	85.5	54	96.5
3	U(2,6)	(1,1,− 1)	71.0	70.5	17.0	58.0	67.0	50.5	85.5
3	U(2,6)	(1,− 1,− 1)	70.0	68.5	17.0	65.5	63.5	52.3	87.5
3	N(3,4)	(1,1,1)	82.5	60.0	83.0	74.0	87.0	71.8	94.0
3	N(3,4)	(1,1,− 1)	68.0	69.0	32.5	72.0	69.0	68.6	82.0
3	N(3,4)	(1,− 1,− 1)	65.5	60.5	32.0	76.0	73.0	67.8	86.5
3	(4,2,1)	(1,1,1)	64.0	3.5	68.5	13.5	78.5	7.9	95.0
3	(4,2,1)	(1,1,− 1)	18.0	3.0	18.5	12.5	31.5	16.1	79.0
3	(4,2,1)	(1,− 1,− 1)	3.0	4.0	0.5	12.0	8.5	9.0	76.0
3	(8,4,2)	(1,1,1)	99.0	98.0	99.5	98.5	98.5	96.5	99.5
3	(8,4,2)	(1,1,− 1)	97.0	96.5	98.5	94.5	98.5	93.9	99.0
3	(8,4,2)	(1,− 1,− 1)	96.0	96.0	0.5	94.0	97.0	87.5	98.0
3	(4,4,2)	(1,1,− 1)	69.0	45.0	45.0	28.5	65.6	43.5	93.5
3	(2,5,4)	(1,− 1,− 1)	92.5	74.5	76.0	65.5	62.0	79.3	99.5
4	(4,4,2,1)	(1,1,1,1)	97.0	47.5	98.5	24.5	98.5	53.2	99.5
4	(4,4,2,1)	(1,1,1,− 1)	95.5	47.0	95.5	24.5	97.5	50.9	99.0
4	(4,4,2,1)	(1,1,− 1,− 1)	71.0	45.0	0.5	24.0	70.5	52.2	93.0
5	(4,4,4,2,1)	(1,1,1,1,1)	100.0	84.5	99.5	35.5	99.5	87.2	100.0
5	(4,4,4,2,1)	(1,1,1,1,− 1)	99.5	84.0	99.0	35.0	98.5	82.1	99.5
5	(4,4,4,2,1)	(1,1,1,− 1,− 1)	98.0	84.0	99.0	34.0	98.5	81.7	99.5

The comparison methods sum test(ST), squared sum test (S2T) and adaptive test (AT) mentioned in the paper are equivalent to the methods burden test ($L_B$), the sum of squared score test ($S_Q$) and the combination of $L_B$ and $S_Q$, respectively. Let $S_S$ denote the score test and $L_W$ denote the weighted sum statistic. The proposed combination method OWC is a combination of $L_B$, $L_W$, $S_S$, and $S_Q$. sumSTAAR is a fexible framework for gene-based association studies using GWAS summary statistics

### Real data analysis

#### Schizophrenia GWAS summary data application

We further evaluated the performance of the proposed method OWC by applying it and the other six methods to two SCZ summary datasets [22]. The two datasets were downloaded from the website of the Psychiatric Genomics Consortium (PGC) (URL https://www.med.unc.edu/pgc/results-and-downloads). The first dataset is a SCZ meta analysis GWAS dataset (13,833 cases and 18,310 controls), denoted as SCZ1 [23]. The second dataset is a more recent study including 36,989 cases and 113,075 controls, denoted as SCZ2 [24]. The MAF, estimated effect size, odds ratio, and *p*-value for 560,833 SNPs on 17,866 genes are included in SCZ1. Similar information for 557,511 SNPs on 17,824 genes are included in SCZ2. Following Wu et al. [25], a gene was defined by including all of the SNPs from 20 kb upstream to 20 kb downstream of the gene. Using OWC and other six tests, we tested the association between the gene and the trait. The 1000 Genomes Project reference panel [18] was used to estimated the LD of pairwise SNPs within each gene of the two datasets. To make fair comparisons among the seven tests, we removed rare variants with MAF< 0.05 and kept one of a pair of SNPs with the coefficient of pairwise LD $r^2>0.5$. After SNPs pruning in quality control, 174,648 SNPs on 17,467 genes in SCZ1 data and 174,275 SNPs on 17,420 genes in SCZ2 data are remained in our final analysis. We used 10^6^ times of Monte Carlo simulation to estimate the *p*-values for the OWC and aSPU method. The Bonferroni corrected significance level $\approx 2.80\times 10^{-6}$ was employed to claim significance in a genome-wide gene-based association study. We first conducted a GWAS for the SCZ1 data [20,899 individuals] with the OWC and the other comparable methods to identify genes associated with SCZ. We then detected genome-wide significant genes associated with SCZ based on the larger SCZ2 dataset [150,064 individuals] which can be considered as a partial validation study for the GWAS based on the SCZ1 data.

**Fig. 1:**
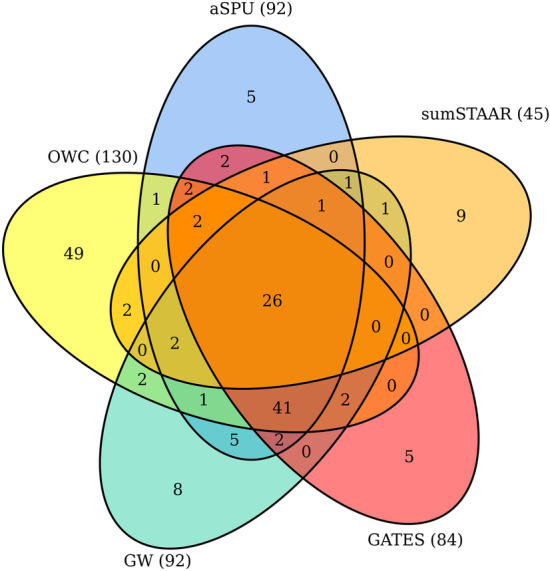
Venn diagram of the number of significant genes identified by OWC, aSPU, GATES, sumSTAAR and GW for SCZ1

Figure 1 shows a Venn diagram of the number of significant genes identified in SCZ1 by the proposed method OWC, aSPU, GATES, sumSTAAR and GW. GW represents the aggregation of genes identified by S2T, ST and AT. The OWC identified the most significant genes (130 genes). sumSTAAR identified the least significant genes (45 genes). Both aSPU and GW identified 92 significant genes. GATES identified 84 significant genes. Among the 130 genes identified by OWC, 78 (i.e. 60%) contained genome-wide significant SNPs (*p*-value 5 × 10^−8^) within 20 kb in the SCZ1 data and 86 (around 66.2%) contained genome-wide significant SNPs within 20 kb in the SCZ2 data. This offered significant validation of the identified genes. Thus, our method identified more SCZ associated genes than the other methods. More interestingly, OWC uniquely identified 49 genes in the SCZ1 data and 104 genes in the SCZ2 data. These genes were missed by other methods. Among the 49 genes, 10 genes contained the genome-wide significant SNPs within 20 kb in the SCZ2 data. These identified genes containing highly significant SNPs gave credence to the power and validity of OWC. Overall, we identified 76 significant and unique genes in the SCZ1 data with all these tests. Additional file 2: Significant genes identified by OWC, aSPU, GATES, sumSTAAR, and GW in SCZ1 data, SCZ2 data, and UKB data. Tables S1 and S2 shows information about the significant genes identified by OWC, aSPU, GATES, sumSTAAR and GW in SCZ1 data and SCZ2 data, respectively.

**Fig. 2:**
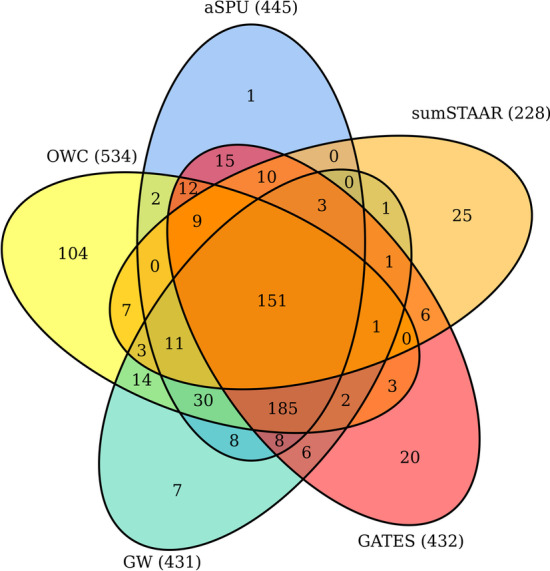
Venn diagram of the number of significant genes identified by OWC, aSPU, GATES, sumSTAAR and GW for SCZ2

Next, the seven tests were applied to the SCZ2 data. Figure 2 shows the numbers of significant genes identified by OWC, aSPU, GATES, sumSTAAR and GW. Similarly, the OWC identified the most significant genes (534 genes). Among the 534 genes, 398 genes (74.5%) contained genome-wide significant SNPs (*p*-value 5 × 10^−8^) within 20 kb in the SCZ2 data. sumSTAAR identified the least significant genes (228 genes). GATES identified 432 significant genes. GW identified 431 significant genes, similarly, aSPU identified 445 genes. As expected, all the methords identified more significant genes in the SCZ2 data than in the SCZ1 data since the sample size of the SCZ2 dataset is much larger than that of SCZ1 [22]. Again, our method OWC is more powerful than the other methods in terms of the total number of significant genes being identified. We further noticed that each of these tests identified some unique genes but missed by the others. This suggests that different tests may be powerful in different scenarios. In the SCZ2 data, OWC identified 104 significant and unique genes (Additional file 2: Significant genes identified by OWC, aSPU, GATES, sumSTAAR, and GW in SCZ1 data, SCZ2 data and UKB data. Table S2 shows information about significant genes identified by OWC, aSPU, GATES, sumSTAAR, and GW in SCZ2 data).

The computational time of OWC in a genome-wide association study is acceptable, though the Monte Carlo simulation method is employed to estimate the *p*-value of OWC. For example, there are 17,467 genes in the SCZ1 GWAS summary data. We used 10^6^ times of simulations to estimate the *p*-value of OWC. The computational time of *p*-value estimation of OWC for a gene based on 10^6^ simulations is about 20 minutes when we use the R package of OWC with the fast algorithm [25] on a Dell PowerEdge C6320 server which includes two 2.4 GHz Intel Xeon E5-2680 v4 fourteen-core processors with average memory as 600 MB. The estimated time for completing a whole genome-wide association study for the 17,467 genes would be less than a day if we run the jobs on 500 such servers concurrently.

#### T2D GWAS summary data application

Furthermore, we performed a comprehensive study for fasting glucose in a type 2 diabetes (T2D) GWAS summary data obtained from the UK Biobank component of the European DIAMANTE study (denoted as UKB). It included over 440,000 individuals [19,119 cases and 423,698 controls] of European ancestry. This GWAS using the UK Biobank Resource under Application Number 9161 (McCarthy) was restricted to HRC variants. We downloaded the GWAS summary data from http://www.type2diabetesgenetics.org/informational/data. The UKB summary data consists of information about MAF, estimated effect size, odds ratio, and *p*-value for approximately 17,850 genes [27]. The same filtering and analyzing procedure used in the SCZ data was employed in the UKB data. The significance level $0.05/17,850\approx 2.80\times 10^{-6}$ was used in this study. We performed 10^6^ simulations to estimate *p*-values for the OWC and aSPU method.

**Fig. 3:**
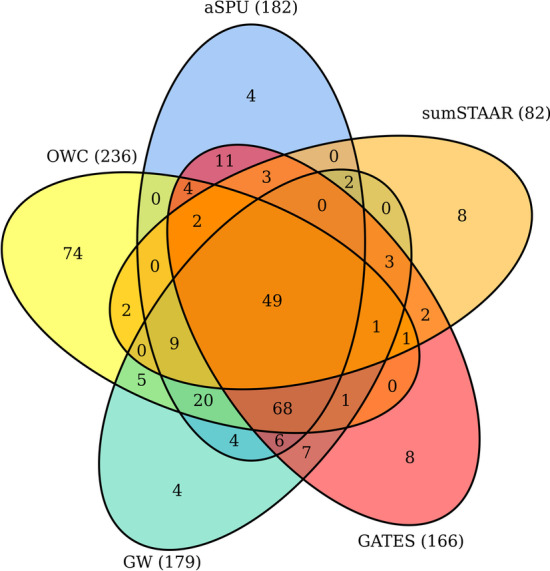
Venn diagram of the number of significant genes identified by OWC, aSPU, GATES, sumSTAAR and GW for UKB

The Venn diagram in Fig. 3 shows the number of significant genes identified by OWC, aSPU, GATES, sumSTAAR and GW, respectively. The OWC identified 236 significant genes which is much larger than the number of genes identified by the other methods (aSPU [182 genes], GATES [166 genes], sumSTAAR [82], and GW [179 genes]). Around 41.1% [97 out of 236] of the significant genes identified by OWC contained the genome-wide significant SNPs (*p*-value < 5 × 10^−8^) within 20 kb in the UKB data [27]. Based on the number of significant genes identified in the UKB data, we can further conclude that the proposed OWC method performed the best compared to the other tests. Additional file 2: Significant genes identified by OWC, aSPU, GATES, sumSTAAR, and GW in SCZ1 data, SCZ2 data and UKB data. Table S3 shows the information about the significant genes identified by all the methods in the UKB data.

## Discussion

Weighting genetic variants in a gene appropriately plays an important role to boost the power of a gene based association test. In this paper, we propose a novel combination test - OWC. This is a general linear combination test incorporating four different weighting schemes: two constant weights and two weights proportional to normal statistics ${\varvec{Z}}$. The burden test, WSS, SSU, and score test are four typical gene-based tests, which are included in the OWC as its special cases. When we focus on rare variants analysis summary data, the elements on the diagonal of matrix $\varvec{A}$ can be estimated from the beta distribution with pre-specified shape parameters in its density function as 1 and 25. In this situation, the method SSU contained in OWC is the SKAT method. Therefore, we can view the SKAT and SKAT-O methods as special cases of the proposed method. When we have data from transcriptome-wide association studies, we can set the elements of the diagonal of matrix $\varvec{A}$ being the estimated cis-effects from gene expression as weights of variants for the WSS. Then, the WSS and SSU contained in the proposed OWC method become PathSPU(1) and PathSPU(2) [28]. As a general method with a maximized test statistic, the OWC can reach the largest power.

Furthermore, we show that the general linear combination test statistic can reach its maximum when the weight is estimated as a certain value. For example, the score test $S_S$ , as a special case of OWC, reaches its maximum when the weight is the product of the inverse of the correlation matrix $\varvec{R}$ among SNPs and Z-scores. A correct estimation of the correlation matrix $\varvec{R}$ is critical. To alleviate the errors in estimating $\varvec{R}$, Deng and Pan (2018) proposed an estimator of the correlation matrix $\varvec{R}$. Their idea is similar to multiple imputation [29]. In real studies, we suggest to remove low frequency (e.g. $\hbox {MAF}<0.05$) variants and one of a pair of SNPs with pairwise LD $r^{2}$ greater than a prespecified threshold. We tested OWC on ten genes based on a real SCZ1 data and the estimated $\rho _3$ was always larger than 0.5. In this case, the score test may make the main contribution in the power of OWC. When the correlation among SNPs is ignored (i.e. $\varvec{R}={\varvec{I}}$), OWC becomes the SSU test. ST, S2T, AT, aSPU, GATES, and sumSTAAR are the most popular existing methods using GWAS summary data. We compared the performance of the proposed test OWC with the six comparison methods in both simulation studies and real data analyses. Extensive simulation studies demonstrate that the proposed test OWC is not only valid but also powerful in most of the scenarios. In real data analyses, OWC identifies the largest number of disease associated genes compared to the other comparison methods.

True disease model is usually unknown. Disease models underlying different diseases may be different: some of the disease models may include causal genetic variants with same directions while other disease models may include causal genetic variants with different directions. In addition, some diseases models may include some weakly associated genetic variants, while other disease models may include some strongly associated genetic variants. There is no uniformly most powerful test that is powerful in every situation. An association test may perform well in one dataset, but may perform less well in another dataset. For example, SCZ can be considered as a representative of complex disease. People have identified some common genetic variants with weak effects on SCZ. These variants may be working in tandem to produce SCZ. A robust, flexible method such as OWC can elucidate these weakly associated genetic variants better so that the roles of theses genetic variants in disease etiology can be understood more clearly. The proposed OWC method can be a useful tool as it adapts to the underlying biological disease model for a disease by selecting $\varvec{\rho }$ based on the data.

In summary, the novelty of the proposed method lies in two aspects: 1) proposing a new score test $S_s$ which reaches its maximum through finding the certain weights for genetic variants; 2) proposing a new combination method OWC which reaches its maximum through finding certain weights for the combination of the four component tests. Also, the score test is a component of OWC. Through using two types of optimizations, the OWC is more powerful than other comparison methods in most situations which is demonstrated in Table 3 on the manuscript.

The proposed OWC method only needs the publicly available GWAS summary statistics as input, without the need to access raw genotype and phenotype data. Researchers will be able to identify more novel disease associated genes with OWC by utilizing publicly available GWAS summary data. Novel disease associated genes can shed more light onto underlying mechanism of diseases. In this paper, we focus on developing a powerful genetic associated test using single trait GWAS summary data. The proposed OWC method can be easily extended to analyze GWAS summary data for multiple traits. We have implemented OWC in an R package which is freely available at https://github.com/Xuexia-Wang/OWC-R-package.

## Methods

### Expressing gene based methods with a weighted combination of Z-scores

Consider a sample including *n* individuals with both genotype and phenotype data available in a genomics region (gene or pathway) with M genetic variants (e.g. SNPs). For the $i^{th}$ individual, denote $y_i$ as the trait value which is either a quantitative or qualitative trait (1 for cases and 0 for controls), denote $X_i = (x_{i1},\ldots ,x_{iM})^{'}$ as the genotypic score for the considered region, where $x_{im}\in \{0, 1, 2\}$ is the number of minor alleles at the $m^{th}$ variant. $x_{im}$ can also be the number of minor alleles in dominant, recessive coding, or imputed dosage that the $i^{th}$ individual has at the $m^{th}$ variant. Although the formulas derived in the Methods section is based on genotypes with additive coding, the conclusions are still held when the genotypes are centered with mean 0.

The generalized linear model was used to model the relationship between the trait and the genetic variants in the considered region:

$$\begin{aligned} f(E(y_i|X_i))=\beta _0+\varvec{\beta _c}^{'}X_i \end{aligned}$$

where $f(\cdot )$ is a monotone “link” function and the vector $\varvec{\beta _c}$ is the parameter of interest which represents the fixed effects of the genetic variants. Testing the association between the genetic variants in the region and the trait is equivalent to test the effect of the weighted combination of genetic variants $g_i=\sum _{m=1}^Mw^0_mx_{im}$ on the trait. Under the generalized linear model, we propose to use the score test statistic [30] to test the null hypothesis $H_0: \varvec{\beta _c}=0$. The score statistic can be expressed as follows:

$$\begin{aligned} \varvec{S}(w^0_1,\ldots ,w^0_M)=n\frac{\big (\sum _{i=1}^n(y_i-{\bar{y}})(g_i-{\bar{g}})\big )^2}{\sum _{i=1}^n(y_i-{\bar{y}})^2\sum _{i=1}^n(g_i-{\bar{g}})^2} \end{aligned}$$

The score test statistic $\varvec{S}$ can be viewed as a function of weight $\varvec{W_0}=(w^0_1,\ldots ,w^0_M)^{'}$. Let $Y=(y_1,\ldots ,y_n)^{'}$, $\varvec{X}=(X_1,\ldots ,X_n)^{'}$. Denote $P=I_n-\frac{1}{n}1_n1_n^{'}$, where $1_n$ represents a column vector containing all ones. *P* is an idempotent matrix. That is, $P=P^{'},PP=P$. Considering $x_i=X_i^{'}\varvec{W_0}$, we can rewrite the score test as:

$$\begin{aligned} \varvec{S}(w^0_1,\ldots ,w^0_M)&=n\frac{\varvec{W_0}^{'}\varvec{X}^{'}PYY^{'}P\varvec{XW_0}}{\varvec{W_0}^{'}\varvec{X}^{'}P\varvec{X}Y^{'}PY\varvec{W_0}} \end{aligned}$$

Detailed derivation of the aforementioned score statistic can be found in the supplementary materials (Additional file 1: Derivation of the score test). When real genotype and phenotype data are available, the score statistic can be maximized and extended to a General method to Test the effect of the Optimally Weighted combination of genetic variants in a gene (G-TOW) [30].

To test the association between a trait and a genetic variant, a Z test is usually employed. We can use the Z test below to test the main effect of the $m^{th}$ variant in the considered region on the trait. $Z_m=\frac{Y^{'}PX_{m\cdot }}{\sigma \sqrt{X_{m\cdot }^{'}PX_{m\cdot }}}$ where $\sigma =\sqrt{\frac{1}{n}Y^{'}PY}$ and $X_{m\cdot }=(x_{m1},\ldots ,x_{mn})^{'}$.

Denote the linkage disequilibrium (LD) matrix for the considered region as $\varvec{R}=diag(\varvec{D})^{-1/2}\varvec{D} diag(\varvec{D})^{-1/2}$, where $\varvec{D}=\varvec{X}^{'}P\varvec{X}$ and $diag(\varvec{D})$ denotes the diagonal matrix of $\varvec{D}$. When GWAS summary statistics such as the Z-scores and the LD matrix for genetic variants in the considered region are available, the score statistic can be written as:

1 $$\begin{aligned} \varvec{S}(w_1,\ldots ,w_M)&=\frac{\varvec{W}^{'}\varvec{Z}\varvec{Z}^{'}\varvec{W}}{\varvec{W}^{'}\varvec{R}\varvec{W}} \end{aligned}$$

where $\varvec{Z}=(Z_1,\ldots ,Z_M)^{'}$ and $\varvec{W}=(w_1,\ldots ,w_M)^{'}=diag(\varvec{D})^{1/2}\varvec{W_0}$ (see Additional file 1: Derivation of the score test). From equation (1), the score statistic $\varvec{S}$ is equivalent to a linearly weighted test statistic based on Z-scores:

2 $$\begin{aligned} \varvec{L}(w_1,\ldots ,w_M)=\sum _{m=1}^Mw_mZ_m=\varvec{W}^{'}\varvec{Z} \end{aligned}$$

Under the null hypothesis, ${\varvec{Z}}$ follows multivariate normal distribution with mean ${\varvec{0}}$ and covariance matrix $\varvec{R}$ [31]. This conclusion clearly demonstrates that testing the weighted combination of genetic variants in a considered region using the score test is the same as using the weighted combination of Z-scores for those variants.

In the aforementioned weight function $\varvec{W}=(w_1,\ldots ,w_M)^{'}$, the true value of each weight is unknown and must be determined biologically or empirically. Therefore, in real data analysis, we should give reasonable values of weights in advance for a gene-based test. If all or most of the genetic variants in the region have almost an equal effect size in the same direction of association, we set $w_m=1$ for $m=1,\ldots ,M$, and the test becomes the burden test $\varvec{L}_B=\varvec{L}(1,\ldots ,1)$, which sums up the association signals across all the variants and obtains high power. If we believe that the causal genetic variants would be subject to “purifying selection” and thus appear less frequently in the population than neutral variants, we set $w_m=1/\sqrt{p_m(1-p_m)}$, where $p_m$ denotes MAF of the $m^{th}$ variant, and obtain $\varvec{L}_W=\varvec{L}(1/\sqrt{p_1(1-p_1)},\ldots ,1/\sqrt{p_M(1-p_M)})$, which is the weighted sum statistic (WSS) [12]. If we assume that the values of the weights $\varvec{W}$ come from gene expression or functional annotation data, the test degenerates into the PathSPU(1) test [28]. We know that $\varvec{S}(w_1,\ldots ,w_M)$ follows central chi-square distribution with 1 degree of freedom ($\chi ^2_1$) and $\varvec{L}(w_1,\ldots ,w_M)$ follows multivariate normal distribution with mean ${\varvec{0}}$ and covariance matrix $\varvec{W}^{'}\varvec{R}\varvec{W}$ under the null hypothesis, given the choice of the weight function $\varvec{W}$ is not proportional to ${\varvec{Z}}$.

As a function of $\varvec{W}=(w_1,\ldots ,w_M)^{'}$, either the score test $\varvec{S}(w_1,\ldots ,w_M)$ or the linear weighted test statistic $\varvec{L}(w_1,\ldots ,w_M)$ can reach its maximum when we choose an appropriate weight $\varvec{W}$. According to conclusions in Li and Lagakos [32], we have

$$\begin{aligned} \sup _{\varvec{W}}\{\varvec{S}(w_1,\ldots ,w_M)\}&= \sup _{\varvec{W}}\left\{ \frac{L(w_1,\ldots ,w_M)^2}{{\textrm{Var}}(L(w_1,\ldots ,w_M))}\right\} \\&=\sup _{\varvec{W}}\left\{ \frac{\varvec{W}^{'}\varvec{Z}\varvec{Z}^{'}\varvec{W}}{\varvec{W}^{'}\varvec{R}\varvec{W}}\right\} \\&=\varvec{Z}^{'}\varvec{R}^{-1}\varvec{Z} \end{aligned}$$

When $\widehat{\varvec{W}}=\varvec{R}^{-1}\varvec{Z}$, the score test statistic $\varvec{S}(w_1,\ldots ,w_M)$ reaches its maximum value. Given the asymptotic null distribution of ${\varvec{Z}}$ in Eq. (2), we define the score test

3 $$\begin{aligned} \varvec{S}_S=\widehat{\varvec{W}}^{'}\varvec{Z}=\varvec{Z}^{'}\varvec{R}^{-1}\varvec{Z} \end{aligned}$$

which follows central chi-square distribution with *M* degrees of freedom ($\chi _M^2$). The appropriate weights can be obtained when the linear weighted test statistic reaches its maximum value [33]. Although $S_S$ may not have high power when its degree freedom is large, it gives higher weights to the SNPs that have weak correlation with other SNPs. When the correlation matrix $\varvec{R}$ of ${\varvec{Z}}$ is a diagonal matrix denoted as $\varvec{A}=diag(a_1,\ldots ,a_M)$ where $0< a_i\le 1$, that is, $\varvec{R}=\varvec{A}$, we have $\varvec{W}=\varvec{A}^{-1}\varvec{Z}$. The score test in Equation (1), which is equivalent to the linear weighted test in Equation (2), will reach its maximum value when $\varvec{W}=\varvec{A}^{-1}\varvec{Z}$.

To test the association between genetic variants in a considered region and a trait, Kwak and Pan [6] proposed a class of approaches called sum of powered score (SPU) tests along with its data-adaptive version (aSPU), $SPU(\gamma )=\sum _{m=1}^{M}{Z}_{m}^{\gamma }$ and $\gamma =1,2,\ldots ,8,\infty$. The SPU method can also be viewed as a special combination test method with weight $\varvec{W}=\varvec{Z}^{\gamma -1}$. aSPU can be viewed as a data-adaptive weighted combination test method.

When the diagonal matrix *A* is the identity matrix $\varvec{A}={\varvec{I}}$, we denote the test in Equation (3) as $S_Q=\varvec{Z}^{'}\varvec{Z}$, which is the same as the sum of squared score test (SSU) [34] and the variance component test [35]. Based on the asymptotic null distribution of ${\varvec{Z}}$ in Equation (2), the test $S_Q=\varvec{Z}^{'}\varvec{Z}$ follows a mixture of chi square distribution under the null hypothesis: $S_Q\sim \sum _{m=1}^M\lambda _m\chi ^2_1$, where $\lambda _1,\ldots ,\lambda _M$ are the eigenvalues of $\varvec{R}$. Particularly, if we set the diagonal element of $\varvec{A}$ as the beta distribution density function with pre-specified shape parameters as 1 and 25, which are evaluated at the corresponding sample MAF in the data, the score test degenerates into the sequence kernel association test (SKAT) for rare variants [13]. If the value of the diagonal elements $\varvec{A}$ comes from a set of gene expression derived weights, the score test degenerates into PathSPU(2) test method [28]. Naturally, these two methods (SKAT and PathSPU(2)) both follow a mixture of chi square distribution under the null hypothesis. In our paper, we only consider GWAS summary data for common variants, so we set $\varvec{A}$ as the identity matrix for this case.

### A new gene-based method

We have proved and demonstrated that most of the gene-based associate tests can be expressed as a weighted combination of Z-scores. Thus, we can propose a new weighted combination method by utilizing the good properties of different weights. The statistics of $L_B, L_W, S_S$, and $S_Q$ represent four typical weighted methods. To combine the strength of $L_B, L_W, S_S$, and $S_Q$, we consider their weighted average:

$$\begin{aligned} L_{{\varvec{\rho }}}&=\rho _1(L_B)^2+\rho _2(L_W)^2+\rho _3S_S+\rho _4S_Q\\&=\varvec{Z}^{'}\varvec{A}\varvec{Z} \end{aligned}$$

where $\varvec{A}=\rho _1\varvec{1}\varvec{1}^{'}+\rho _2\varvec{W}\varvec{W}^{'}+\rho _3\varvec{R}^{-1}+\rho _4{\varvec{I}}$, $\varvec{1}$ denotes a column vector containing all 1s, $\rho _1+\rho _2+\rho _3+\rho _4=1$, and $0\le \rho _i\le 1$ for $i=1,2,3,4$. Under the null hypothesis, for a given $\varvec{\rho }$, $L_{{\varvec{\rho }}}$ is a linear combination of independent central $\chi _1^2$ random variables:

$$\begin{aligned} L_{{\varvec{\rho }}}\sim \sum _{i=1}^M\lambda _i\chi _1^2 \end{aligned}$$

where $\chi _1^2$ denotes a central $\chi ^2$ random variable with 1 degree of freedom and $\lambda _i$ for $i=1,\ldots ,M$ are the eigenvalues of $\varvec{RA}$ [4]. We propose a novel method - OWC. For a set of values of $\varvec{\rho }$, OWC test can be achieved by using the minimum *p*-value across the values of $\varvec{\rho }$:

4 $$\begin{aligned} T=\min _{\varvec{\rho }}p_{L_{\varvec{\rho }}} \end{aligned}$$

where $p_{L_{\varvec{\rho }}}$ is the estimated *p*-value of $L_{\varvec{\rho }}$. Naturally, *T* can be obtained by a simple grid search across a range of $\varvec{\rho }$: $\{\rho _1,\rho _2,\rho _3,\rho _4\}$. The test statistic $T=\min \{p_{L_{{\rho }_1}},\ldots ,p_{L_{{\rho }_4}}\}$. We search over $\rho _i\in (0,0.1,0.2,0.3,0.4,0.5,0.6,0.7,0.8,0.9,1)$ for $i=1,2,3,4$. Specifically, if $\rho _2=\rho _3=0$ in $\varvec{\rho }$, $L_{{\varvec{\rho }}}$ can be rewritten as $\rho _1(L_B)^2+(1-\rho _1)S_Q$, which is equivalent to SKAT-O test method [14].

### *p*-value estimation

Monte Carlo simulations are used to obtain the *p*-values for *T* in a single layer of simulations. Briefly, after obtaining $\varvec{R}$, we first simulate null scores of ${\varvec{Z}}^{(b)}\sim N(0, \varvec{R})$ for $b=1,\ldots , B$. Then, we use the null scores to calculate the null test statistic $T^{b}$ following the aforementioned procedure for each b, and then the *p*-value of the test is the proportion of the number of the null test statistic $T^{b}$ with $T^{b}\le T$ [36]. A larger B is needed to estimate a smaller *p*-value.

The aforementioned vector ${\varvec{Z}}^{(b)}$ can be generated in the following way [37]: we first generate a vector $\varvec{L}$ with *M* elements where each element is independently generated from a standard univariate normal distribution with mean 0 and variance 1; that is, $\varvec{L}\sim N({\varvec{0}}, {\varvec{I}})$. We then have ${\varvec{Z}}^{(b)}=\varvec{D}\varvec{L}$, where $\varvec{D}$ is obtained from Cholesky decompositions of $\varvec{R}$ with $\varvec{R}=\varvec{D}\varvec{D}^{'}$. Specifically, for the test statistic $T({\varvec{Z}}, \varvec{R})$ as a function of ${\varvec{Z}}$ and $\varvec{R}$, we can estimate its *p*-value in detail as follows:

1. Generate independent ${\varvec{Z}}^{(b)}\sim N(0, \varvec{R})$ for $b=1,\ldots ,B$.
2. Using asymptotic distribution of $L_{{\varvec{\rho }}}$ under null hypothesis, calculate the null test statistic *T* by searching across a range of $\varvec{\rho }$ for ${\varvec{Z}}$ and ${\varvec{Z}}^{(b)}$, respectively.
3. Finally, the *p*-value for the *T* test, $p_{T}$, is
  5 $$\begin{aligned} p_{T}=\bigg [\sum _{b=1}^BI\big (T({\varvec{Z}}^{(b)}, \varvec{R})\le T({\varvec{Z}}, \varvec{R})\big )+1 \bigg ]/(B+1) \end{aligned}$$
  where $T({\varvec{Z}}, \varvec{R})$ is the value of *T* test based on the observed data, $T({\varvec{Z}}^{(b)}, \varvec{R})$ is the value of *T* test based on the $b^{th}$ sampling data.

If the Z statistic in the summary data is not provided, we need to first transform the *p*-value in the summary data into a Z statistic using $Z=\textrm{sign}(\beta )\Phi ^{-1}(1-p/2)$, where $\Phi$ is the cumulative distribution function of the standard univariate normal distribution. Then, a similar procedure can be used to obtain the *p*-value of the test *T*.

One limitation of the Monte Carlo simulation to estimate *p*-values, such as the above one, is the computational burden. Especially, when there are about twenty thousands genes in a GWAS and a small significance level is used to claim significant findings. We adopted a fast algorithm [26] to estimate *p*-values, which will dramatically reduce the computational time. This algorithm reduces computational time by scarifying the precision of the *p*-value estimation for those tests with large true *p*-values.

We first define the following parameters for the algorithm:

- $$B_{max}= \hbox {maximum number of random sampling}$$ (e.g.$$10^6$$)
- $$B_0= \hbox {minimum number of random sampling}$$ (e.g. 10)
- $$p_0=\hbox {a constant }\times \,\hbox {significance level}$$ (e.g.$$5 \times 10^{-6}$$)
- *M* = multiplying increment for the number of random sampling (e.g. 10)
- The fast algorithm works as follows:
  - *Step 0* Calculate the statistic *T* of OWC based on the observed data
  - *Step 1* Set initial values: $p_0=10^{-5}$, $B_{max}=10^6$, $B_0=10$, $M=10$, $B=B_0$
  - *Step 2* Use Eqs. (4) and (5) to estimate *p*-value, ${\hat{p}}$. Let $B=B \times M$
  - *Step 3* If ${\hat{p}}$>$p_0$ or $B>B_{max}$, report ${\hat{p}}$ and stop; otherwise go to step 2.

## Conclusions

Current gene-based association tests, while providing greater interpretability and power over usual single variant association tests, still have many limitations such as weights predetermined biologically or empirically. In this paper, we propose a combination test OWC to overcome these limitations. OWC is a general linear combination test which uses GWAS summary statistics as its input and incorporates different weighting schemes, and includes traditional gene-based tests as its special cases. Simulation studies and real data analyses demonstrate that OWC is more powerful than comparable methods in many scenarios and can adapt to the (generally unknown) underlying genetic architecture of the trait of interest. While the focus of this paper was single-trait analysis, OWC can be easily extended to analyze GWAS summary data for multiple traits.

## Supplementary Information

**Additional file 1**. Linkage equilibrium matrix of gene EPB41.

**Additional file 2**. Significant genes identified by OWC, aSPU, GATES, sumSTAAR, and GW in SCZ1 data, SCZ2 data and UKB data.

## Data Availability

The GWAS summary data of schizophrenia that was obtained from the Psychiatric Genomics Consortium can be downloaded from https://www.med.unc.edu/pgc/download-results/. The GWAS meta-analysis summary data for fasting glucose that was obtained from the European DIAMANTE study can be downloaded from https://t2d.hugeamp.org/datasets.html.
