# Supplementary material for: A gene based combination test using GWAS summary data
Source: BMC Bioinformatics. 2023 Jan 3;24:2. doi: 10.1186/s12859-022-05114-x (PMC9811798; doi:10.1186/s12859-022-05114-x)
Supplement: Supplementary file 1 — Additional file 1. Linkage equilibrium matrix of gene EPB41. [file 12859_2022_5114_MOESM1_ESM.pdf]

A Gene Based Combination Test Using GWAS Summary Data

Jianjun Zhang, Xiaoyu Liang, Samantha Gonzales, Jianguo Liu, Xiaoyi Raymond Gao and Xuexia Wang*

*Correspondence: Xuexia Wang, xuexwang@fiu.edu, Professor of Biostatistics, Department of Biostatistics, Florida International University, 11200 SW 8th Street, Miami, FL 33174, USA.

Derivation of the score test

Use the notation in the Method section. Consider a raw data set of a sample including n individuals, where each individual has been genotyped at M variants in a genomic region (gene or pathway). Denote y_i as the trait value of the ith individual for either a quantitative or qualitative trait (1 for cases and 0 for controls for a qualitative trait) and denote $X_i = (x_{i1}, \cdots, x_{iM})'$ as the genotypic score of the ith individual, where $x_{im} \in \{0, 1, 2\}$ is the number of minor alleles that the ith individual has at the mth variant.

We use the generalized linear model to model the relationship between the trait (quantitative or qualitative trait) and the genetic variants in the considered region:

$$f(\mathbb{E}(y_i|X_i)) = \beta_0 + \boldsymbol{\beta_c}' X_i$$

where $f(\cdot)$ is a monotone "link" function and $\boldsymbol{\beta_c}$ is the parameter of interest. Testing the association of the genetic variants in the considered region is equivalent to testing the effect of the weighted combination of variants $g_i = \sum_{m=1}^{M} w_m^0 x_{im}$. Under the generalized linear model, we can use the score test statistic to test the null hypothesis $H_0 : \boldsymbol{\beta_c} = 0$, which is given by

$$\boldsymbol{S}(w_1^0, \cdots, w_M^0) = n \frac{\left(\sum_{i=1}^{n}(y_i - \bar{y})(g_i - \bar{g})\right)^2}{\sum_{i=1}^{n}(y_i - \bar{y})^2 \sum_{i=1}^{n}(g_i - \bar{g})^2}$$

where the score test statistic $\boldsymbol{S}$ can be viewed as a function of weight $\boldsymbol{W_0} = (w_1^0, \cdots, w_M^0)'$. Let $\boldsymbol{X} = (X_1, \cdots, X_n)'$, $Y = (y_1, \cdots, y_n)'$ and $P = I_n - \frac{1}{n} 1_n 1_n'$ where 1_n represents a column vector containing all ones. Then, we have $x_i = X_i' \boldsymbol{W_0}$. We can rewrite the score test as:

$$\begin{aligned}
\boldsymbol{S}(w_1^0, \cdots, w_M^0) &= n \frac{(PY)' P \boldsymbol{X} \boldsymbol{W_0} (PY)' P \boldsymbol{X} \boldsymbol{W_0}}{(PY)' PY (P \boldsymbol{X} \boldsymbol{W_0})' P \boldsymbol{X} \boldsymbol{W_0}} \\
&= n \frac{\boldsymbol{W_0}' \boldsymbol{X}' PYY' P \boldsymbol{X} \boldsymbol{W_0}}{Y' PY \boldsymbol{W_0}' \boldsymbol{X}' P \boldsymbol{X} \boldsymbol{W_0}} \\
&= n \frac{\boldsymbol{W_0}' \boldsymbol{X}' PYY' P \boldsymbol{X} \boldsymbol{W_0}}{\boldsymbol{W_0}' \boldsymbol{X}' P \boldsymbol{X} Y' PY \boldsymbol{W_0}}
\end{aligned}$$

where the second equation holds because we have $P = P'$ and $PP' = P$, and the third equation holds because $Y' PY$ is a constant.

To test the association between a single trait and a single variant, which we refer to as single-nucleotide polymorphism (SNP), we usually employ a Z test. To test the main effect of the mth variant in the considered region, we use the Z test: $Z_m = \frac{Y' P X_{m\cdot}}{\sigma \sqrt{X_{m\cdot}' P X_{m\cdot}}}$ where $\sigma = \sqrt{\frac{1}{n} Y' PY}$

, $X_{m\cdot} = (x_{m1}, \cdots, x_{mn})$. Let LD matrix $\boldsymbol{R} = diag(\boldsymbol{D})^{-1/2}\boldsymbol{D}diag(\boldsymbol{D})^{-1/2}$ where $\boldsymbol{D} = \boldsymbol{X}'P\boldsymbol{X}$ and $diag(\boldsymbol{D})$ denote the diagonal matrix of $\boldsymbol{D}$. When GWAS summary statistics such as the Z-statistics and the LD matrix for SNP-SNP correlations are available, the score test can be written as:

$$
\begin{aligned}
&\boldsymbol{S}(w_1, \cdots, w_M) \\
=&\frac{\boldsymbol{W_0}'diag(\boldsymbol{D})^{1/2}\frac{diag(\boldsymbol{D})^{-1/2}\sqrt{n}\boldsymbol{X}'PY}{\sqrt{Y'PY}}}{\boldsymbol{W_0}'diag(\boldsymbol{D})^{1/2}diag(\boldsymbol{D})^{-1/2}\boldsymbol{X}'P\boldsymbol{X}diag(\boldsymbol{D})^{-1/2}diag(\boldsymbol{D})^{1/2}\boldsymbol{W_0}} \\
&\times \frac{\sqrt{n}Y'P\boldsymbol{X}diag(\boldsymbol{D})^{-1/2}}{\sqrt{Y'PY}}diag(\boldsymbol{D})^{1/2}\boldsymbol{W_0} \\
=&\frac{\boldsymbol{W_0}'diag(\boldsymbol{D})^{1/2}\boldsymbol{Z}\boldsymbol{Z}'diag(\boldsymbol{D})^{1/2}\boldsymbol{W_0}}{\boldsymbol{W_0}'diag(\boldsymbol{D})^{1/2}\boldsymbol{R}diag(\boldsymbol{D})^{1/2}\boldsymbol{W_0}} \\
=&\frac{\boldsymbol{W}'\boldsymbol{Z}\boldsymbol{Z}'\boldsymbol{W}}{\boldsymbol{W}'\boldsymbol{R}\boldsymbol{W}}
\end{aligned}
$$

where $\boldsymbol{Z} = (Z_1, \cdots, Z_M)'$ and $\boldsymbol{W} = (w_1, \cdots, w_M)' = diag(\boldsymbol{D})^{1/2}\boldsymbol{W_0}$. From Equation (1), the score test statistic $\boldsymbol{S}$ is equivalent to a linear weighted test statistic based on Z-scores:

$$
\boldsymbol{L}(w_1, \cdots, w_M) = \sum_{m=1}^{M} w_m Z_m = \boldsymbol{W}'\boldsymbol{Z}
$$

where $\boldsymbol{Z}$ follows multivariate normal distribution with mean $\mathbf{0}$ and covariance matrix $\boldsymbol{R}$ under null hypothesis. This conclusion clearly demonstrates that testing the weighted combination of variants in a considered region using the score test is the same as using the weighted combination of Z-scores for the variants in the considered region.

Supplementary Figure S1. The linkage disequilibrium (LD) matrix with pairwise correlations for the 11 SNPs on gene EPB41.

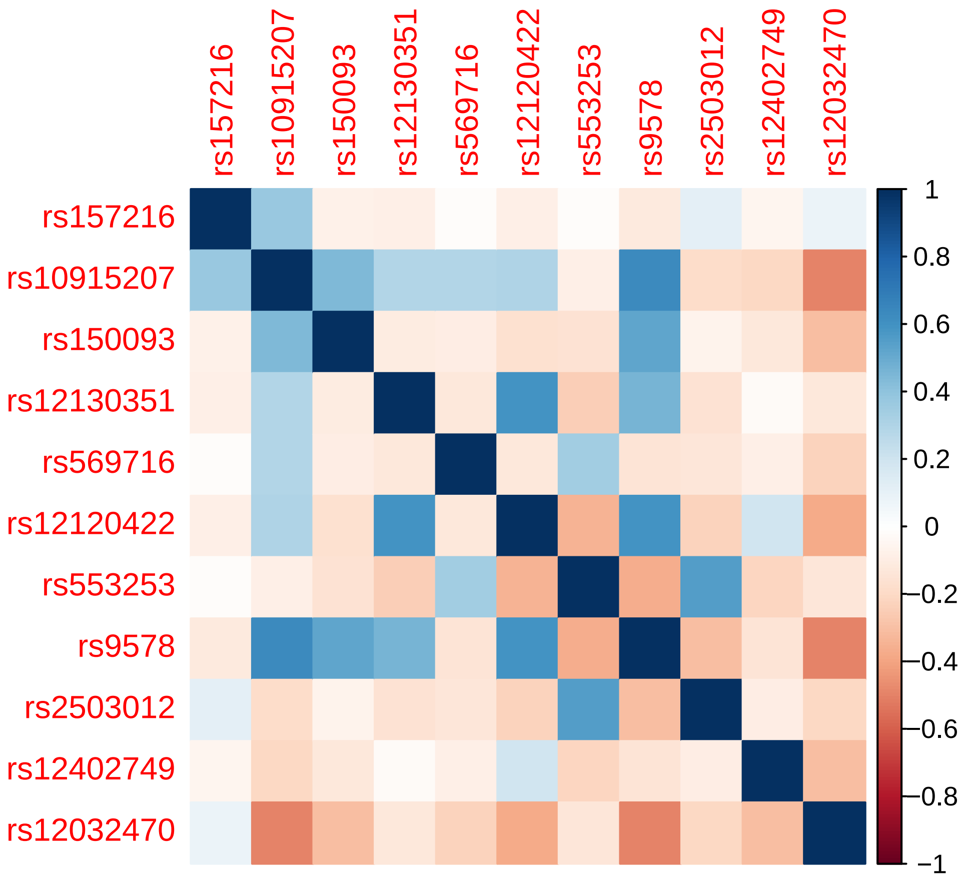

Legends

Supplementary Figure S1. linkage disequilibrium (LD) matrix with pairwise correlations for the 11 SNPs on gene *EPB41*.

Supplementary Table S1. Significant genes identified by OWC, aSPU, GATES, sumSTAAR and GW in SCZ1 data.

Supplementary Table S2. Significant genes identified by OWC, aSPU, GATES, sumSTAAR and GW in SCZ2 data.

Supplementary Table S3. Significant genes identified by OWC, aSPU, GATES, sumSTAAR and GW in UKB data.
